# Sulphide of Calcium for Suppurating Buboes

**Published:** 1882-02

**Authors:** 


### Sulphide of Calcium for Suppurating Buboes.

Professor Otis, following the suggestions of Ringer, employs (*New York Medical Journal*, May, 1880) sulphide of calcium, with the best results, in cases of threatened suppuration in phlegmonous swellings, 0.005 gramme every two hours, or 0.003 gramme every hour during the day. Furuncles and abscesses are arrested; suppurative processes of the mucous membranes, on the contrary, not. Absorption of pus already formed, and resolution of the tumor, occurred in fifteen out of eighteen consecutive cases of inguinal buboes associated with chancroid, this resolution tending to prove (in accordance with the popular teaching) that the buboes were of sympathetic and not of chancroidal origin. Applying to these cases of inflammatory buboes, the concomitants or immediate sequelæ of well-pronounced chancroids, the old rule that chancroidal buboes always eventuate in chancroidal abscesses, always suppurate, and require evacuation, then we must hold that only three out of eighteen cases of inflammatory buboes, associated with chancroids, were the result of transference of the suppurative process from the chancroid to the adjacent lymphatic gland. Or the influecce of the drug may extend to the true chancroidal bubo. At all events its successful use invites a trial of its efficacy in all instances of threatened granular suppuration.—*Boston Medical and Surgical Journal.*

